# *In silico* mining and functional analysis of AP2/ERF gene in *Withania somnifera*

**DOI:** 10.1038/s41598-020-60090-7

**Published:** 2020-03-17

**Authors:** Sandhya Tripathi, Yashdeep Srivastava, Rajender Singh Sangwan, Neelam Singh Sangwan

**Affiliations:** 1 Department of Metabolic and Structural Biology, CSIR-Central Institute of Medicinal and Aromatic Plants (CSIR-CIMAP), Lucknow, 226015 India; 2 Academy of Scientific and Innovative Research (AcSIR) (An Institution of National Importance by Act of Parliament), CSIR-HRDC Campus, Kamla Nehru Nagar, Sector-19, Ghaziabad, 201002 UP India; 3 Department of Biochemistry, School of Interdisciplinary and Applied Life Sciences, Central University of Haryana, Jant-Pali, Mahendergarh, Haryana 123031 India

**Keywords:** Plant molecular biology, Plant stress responses

## Abstract

*Withania somnifera* owing to its strong and remarkable stress tolerance property is a reliable candidate for the determination of genes involved in mechanism of adaption/tolerance of various stress conditions. 187 *AP2/ERF* gene related transcripts (GRTs) were identified during comprehensive search in *W. somnifera* transcriptome repertoire. Major hits in homology search were observed from the model plant *Arabidopsis* and members of Solanaceae family. Cloning, expression analysis of the gene and genetic transient transformation with the gene (*WsAP2*) were performed to predict its functional role *in planta*. Enhanced expression of some of the pathway genes for terpenoid biosynthesis was observed in transformed tissues in comparison to the control tissues. It is speculated that *WsAP2* gene crucially regulates the expression of *GGPPS* gene in addition to the regulation of other important genes of terpenoid pathway *via* induction of expression of other genes such as *HMGR*, *CAS*, *DXS* and *DXR*. To the best of our knowledge, this is the first report representing detailed study of *AP2/ERF* gene family in *W. somnifera*. It is also suggested from the study that gene might have role in eliciting responses to combat stress and attribute the strong stress tolerant property associated with the plant.

## Introduction

Plant transcription factors are involved in the regulation of various aspects of plant growth and development including metabolism, ripening of fruits and defense responses etc. APETALA2/ethylene response factor (AP2/ERF) is a large super family of transcription factors (TF) in plant kingdom, which has been reported to be involved in various cellular processes^1^. AP2/ERF superfamily of transcription factors have been reported to be plant specific however the domain has also been encountered in the proteins associated with cyanobacteria^2^. Classification and structural analysis of AP2 superfamily revealed that the members of this superfamily have AP2 DNA binding domain of 60 amino acids. On the basis of repetitions and sequence of these AP2 domain, the superfamily has been classified in five major subfamilies i.e. AP2, RAV, ERF (Ethylene response factor), DREB (dehydration-responsive element-binding protein) subfamilies and Soloist^3,4^. The ERF and DREB subfamily contains a conserved WLG motif and a single AP2 binding domain while AP2 subfamily has two AP2 domains. The RAV subfamily members have a B3 DNA binding domain in addition to AP2 domain. Other members have single AP2 domain but do not have WLG motif, the characteristic of DREB and ERF subfamily^5^. One twenty one ERF proteins from *Arabidopsis thaliana* were classified in two different groups of DREB and ERF, based on the sequence similarity of their AP2 domain and these two groups have been divided into six subgroups^3^. Further analysis of the exon-intron structure and presence of additional motifs in ERF proteins of *Arabidopsis thaliana* as well as of *Oryza sativa*, has led to division of these proteins into 12 subgroups based on similar regulatory features^6^. The 60 amino acid long AP2 domain of AP2 superfamily is divided into two groups. At N terminal end, 20 amino acid long YRG region is rich in basic and hydrophilic residues that is responsible for binding with DNA element, while 40 amino acid long stretch at C terminal end is known as RAYD region that forms an alpha helix with its 118 amino acid residues. This RAYD region is responsible to mediate protein-protein interactions^7^. The double repeat of AP2 domain in AP2 subfamily is attached by a linker sequence of about 25 amino acids which is highly conserved and important for proper binding of AP2 proteins with their respective DNA elements^8^.

AP2/ERF superfamily genes and related transcripts were previously identified in various plants and extensively studied with respect to stress tolerance^9,10^. Various plants have been examined for genome-wide studies related to AP2 gene in plants such as *Arabidopsis thaliana*^11^, *Ricinus communis L*^12^, *Brassica rapa* ssp. pekinensis^13^, *Vitis vinifera*^14^, *Lotus japonicus*^15^, *Medicago truncatula*^16^, *Populus trichocarpa*^17^, *Musa species*^18^, *Glycine max* L^19^ etc. Further, transcriptomic and EST related studies for AP2 have been carried out in *Brassica* *sp*^20,21^., *Hevea brasiliensis*^22^, *Camellia sinensis*^23^ and *Tricitum aestivum*^24^. Here, in this study, we have identified 187 AP2/ERF GRTs in *W. somnifera* transcriptome. The study presents the first report on identification of AP2/ERF genes in *W. somnifera* in addition to its classification and characterization. Expression of various transcription factors control and regulate the developmental and cellular processes of plants. Therefore, the study will provide a platform for detailed functional analysis of AP2/ERF family genes to serve as resource for understanding the molecular mechanisms associated with stress responses in *W. somnifera*.

One hundred forty five, AP2/ERF genes were characterized in *Arabidopsis* containing 6, 65, 56 and 1 gene representatives belonging to AP2, RAV, ERF, DREB and Soloist respectively. Recent reports on *C. annum* analysed presence of 175 AP2/ERF related transcripts in the latest genome database which were classified as AP2, RAV, ERF, and Soloist members^25^. Similarly, 155 tentative ERF genes were determined in *Solanum tuberosum* using the genome database and the comparison was carried out with *A. thaliana*^26^. Isolation of a cold-inducible protein was proposed to contain AP2/ERF domain and reported in *S tuberosum* previously^27^. Further five ERF genes were also reported to be cloned from *S tuberosum* with predictive roles in hormonal and stress regulation of potato^28^. However, in *W. somnifera* not much information is available on the identification and characterization of the AP2 transcription factor gene, so far.

Putative GRTs encoding for AP2/ERF in *W. somnifera* were used for the identification of total 182 transcripts in the respective superfamily. Phylogenetic as well as protein motif structure analysis was also carried out for AP2/ERF genes. Furthermore, the demonstration of length wise transcript expression in different tissues for these transcripts was also performed. Thus, the functional motif identification and classification into various groups will facilitate further studies related to biological functions for the AP2/ERF family genes in *W. somnifera*.

## Materials and Methods

### Identification of AP2/ERF GRTs in *W. somnifera* through transcriptome analysis

The transcriptome datasets (SRA053485) at NCBI database (https:/www.ncbi.nlm.nih.gov/) and illumina sequenced pooled berry tissue data were used to mine out the AP2 GRTs. GRTs related to AP2/ERF were isolated from *W. somnifera* transcriptome through BLAST (https://blast.ncbi.nlm.nih.gov/Blast.cgi) and annotation analysis. To avoid the redundancy of gene sequences the putative GRTs were assembled further through CAP3 assembler (http://doua.prabi.fr/software/cap3). The sequences were processed and assembled appropriately to ensure dataset having significant transcript length and reduced error rate, as done previously^29,30^. The transcripts screened with appropriate parameters were analyzed using Blast2GO pipeline^31^. The annotations for the GRTs were also confirmed by datasets from AP2/ERF genes at PlantTFDB (http://planttfdb.cbi.pku.edu.cn/) and transcription factor genes at PlantTFcat (http://plantgrn.noble.org/PlantTFcat/).

### Sequence alignment and phylogenetic analysis

The query sequences from *W. somnifera* transcriptome were subjected to multiple sequence alignment along with the the sequences from *S. lycopersicum*, *C. annum* and *N. tabacum* and other plants using tools available (https://www.ebi.ac.uk/Tools). The dataset was then used for tree construction using MEGA 6.06 (https://www.megasoftware.net/). The extent of closeness among the sequences was determined through maximum likelihood using earlier method^32^. Highest log likelihood was applied to construct the tree with branch length obtained through calculation of occurrence of substitution at a particular position.

### Motif analysis

MEME suite, a motif based sequence analysis tool was used for motif identification with average amino acid sequences of upto 25 (http://meme-suite.org/) with a value of 35 as maximum number of motifs allowed for analysis. MEME was used to predict the probability of amino acid at a particular site in the pattern^33^.

### Plant materials

*Withania somnifera* (NMITLI-118) plants were grown the experimental farm and glasshouse of the CSIR- Central Institute of Medicinal and Aromatic Plants, Lucknow, India. Young plantlets of five leaf stage (one month old) were taken for stress and elicitation treatments while mature (six months old) plants were harvested for tissue wide expression analysis and gene isolation studies.

### Isolation of RNA and cDNA synthesis

Flowers of *W. somnifera* (NMITLI-118) were collected and frozen in liquid nitrogen immediately after harvesting. For the isolation of total RNA, TRI reagent (Sigma- Aldrich, US) was utilized following the standard manufacturer’s instructions. The quality of RNA was assesed on 0.8% agarose gel and quantity was checked by spectrophotometric analysis (ND- 1000 Nanodrop, NanoDrop Technologies, US). cDNA was synthesized by using the Revert Aid kit for cDNA synthseis (Fermentas, US) following the manufacturer’s methods.

### Tissue wide transcript quantification studies of *WsAP2*

For relative quantification of levels of transcripts of *WsAP2*, different plant parts (leaf, root, berry and flower) were collected from six months old mature plants of *W. somnifera* and immediately frozen in liquid nitrogen. Further, RNA was isolated and cDNA was synthesized following standard procedure provided by manufacturers (Fermentas) followed by real time PCR analysis using SYBR green master mix (Applied Biosystems). Gene specific real time primers WST11RTF and WST11RTR were used with keeping β-actin gene as an internal reference control. Reactions were carried out using the cycling conditions as described for qRT-PCR above. The ΔΔCt method was used to calculate the expression levels of *WsAP2*^34^.

### Stress and elicitor treatments

One month old plantlets of five leaf stage were employed for various elicitor and stress treatments such as gibberellic acid (GA), methyl jasmonate (MeJA), salicylic acid (SA), wounding, heat-shock and cold. For GA, SA and MeJA treatments, plantlets were dipped in distilled water supplemented with 0.1  mM and 1  mM SA, GA and MeJA separately for 3 hrs, 6hrs, 9hrs, 12hrs, 24 hrs, and 48 hrs, respectively. For heat shock treatment, plantlets were kept at 65 °C for 30  min and 1 hr, for cold treatment plantlets were kept at 4 °C for 30  min and 1  hr. The wound treatment was given by rubbing and puncturing the leaves by sterile syringe, followed by dipping the plantlets in distilled water for 30  min and 1 hr. RNA isolation was done followed by cDNA synthesis and real time transcript quantification of *WsAP2* was carried out by methods described above using same set of primers.

### Cloning of APETALA2/ethylene response factor (AP2-ERF) (*WsAP2*) gene from *W. somnifera*

Full length clone of *WsAP2* (Node_3814) was obtained using cDNA synthesized from RNA of *W somnifera* flowers with gene specific primers. For amplification, Pfu DNA polymerase (Fermentas, US) was used and the PCR thermocyling program started from denaturation at 94 °C for 3  min, followed by 32–35 cycles at 94 °C for 40  sec, 55 °C for 1  min and 72 °C for 2.0  min, and terminated by a final extension step at 72 °C for 10  min. The purified product was then cloned in the pJET1.2 vector and transformed in *E. coli* DH5α cells. Positive clones were screened by colony PCR and used for plasmid isolation. Plasmid was digested by appropriate restriction enzymes and cloned into pBI121 vector containing CaMV 35 S promoter. Positive clones were confirmed by PCR and digestion and was further utilized for transformation in *A. tumefaciens*.

### Transient transformation of *W. somnifera* with *WsAP2-pBI121* construct

Transient transformation was performed to transiently overexpress *WsAP2* transcription factors in *W. somnifera* by the method described in our earlier studies^35^. The cycling parameters were same as described above and β-actin was taken as endogenous control for semi-quantitative PCR. The semi quantitative expression pattern of *WsAP2* was visualized on 0.8% agarose gel. Quantitative real time PCR was performed to validate the results obtained from semi-quantitative PCR and to quantify the *WsAP2* transcript abundance in transformed tissues. qRT-PCR was performed with an ABI PRISM 7500 Real-Time PCR System (ABI, US) with a thermocycling program of 95 °C for 30  sec, followed by 40 cycles of amplification (95 °C for 5  sec, 60 °C for 20  sec, 72 °C for 20  sec). For normalization of expression values, actin of *W. somnifera* was used as a reference gene. All the reactions were conducted with three biological replicates in 10 µl reaction volume.

### GUS assay of putative transformants

*A. tumefaciens* infected putative tissue transformants after transient expression were checked for GUS expression^36^. Briefly, transiently transformed leaves tissues were checked for expression of GUS by utilizing *in-situ* GUS reaction mixture. Tissues were incubated for overnight at 37 °C followed by washing with sterile double distilled water. Further, the tissues were dipped in 70% ethanol for removal of chlorophylls. The tissues were observed under a microscope (Leica EZ4D, Switzerland), and scored for the presence of GUS foci with photo-documentation.

### Transcript abundance studies on secondary metabolite pathway genes in *WsAP2* transformedtissues

The selected genes for this study were CAS (cycloartenol synthase), HMGR (3-hydroxy-3-methylglutaryl coenzyme A reductase), DXS (1-deoxy-D-xylulose-5-phosphate synthase), DXR (1-deoxy-D-xylulose 5-phosphate reductoisomerase) and GGPPS (geranylgeranyl diphosphate synthase). RNA was isolated from wild type and transiently transformed tissues of *WsAP* and cDNA was synthesized. This cDNA was utilized for qRT-PCR analysis as described above. The primer sequences of selected genes are mentioned in Table S1.

## Results

### Mining and annotation of AP2/ERF GRTs

**Figure 1:**
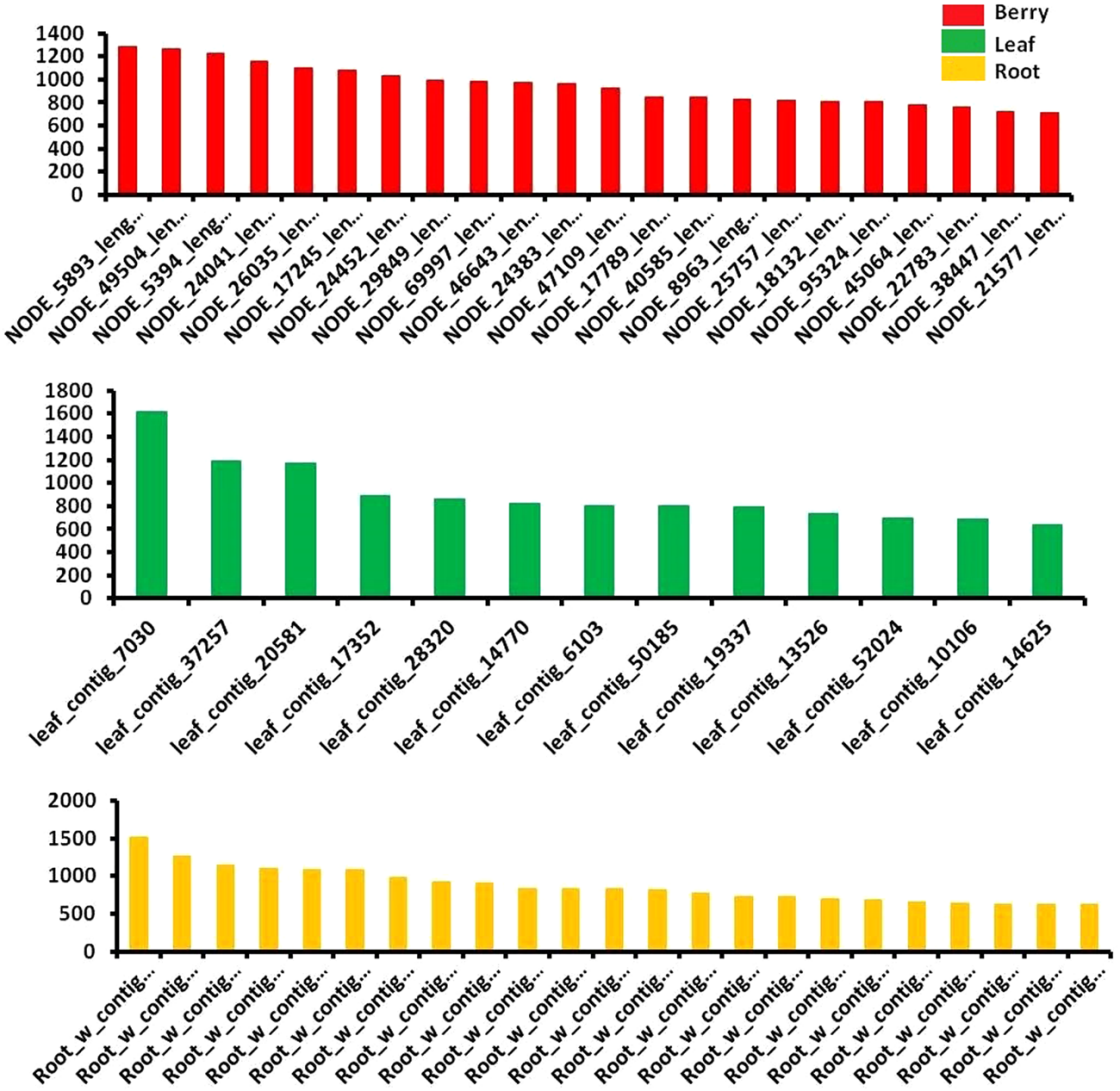
Lengthwise transcript distribution of AP2 GRTs in the three tissues of *W. somnifera*^29,47^.

**Figure 2:**
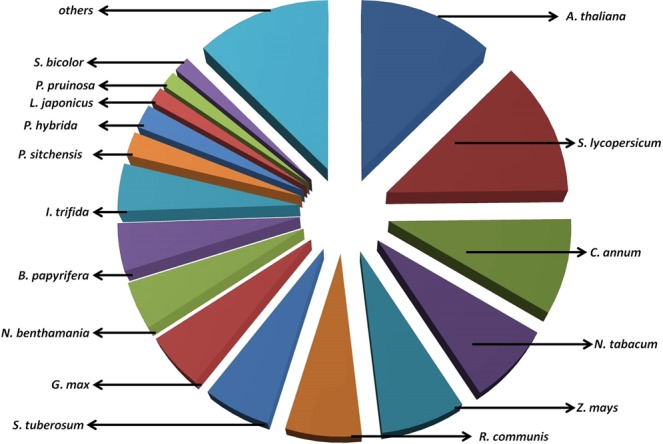
Top hit species distribution for the whole repertoire (combined transcripts from all the transcriptomes) of AP2 GRTs in *W. somnifera* based on annotations obtained through Blast2GO analysis.

Transcripts with significant range of length for AP2/ERF GRTs were observed in the transcriptome (Pooled berry tissue transcripts represented by red bars as various NODE ids and green and yellow bars denote transcripts named as various contig ids for leaf and root tissues respectively, as in our previous reports). Overall 187 AP2/ERF GRTs were found to be present in *W. somnifera* transcriptomes. Significant number of AP2/ERF GRTs of considerable lengths were observed in berry (89), leaf (36) and root (62) tissues during the study which included Blastx analysis for homology search, GO analysis, Interproscan assignment analysis etc. The transcripts having length between 500 to 3000  bp were plotted together for the three tissues (Fig. 1). Top hits were observed for genes reported from *A. thaliana*, various members from Solanaceae family (*S.lycopersicum*, *C. annum*, *N. tabacum*, *S. tuberosum* etc.), *G. max* and various other plants (Fig. 2). The major descriptions for the hits observed in AP2/GRTs were AINTEGUMENTA-like 6, AP2 family protein, ERF family protein, cytokinin response factor, 4, AP2 6 l, ethylene responsive element binding factor2 and so on. 27 transcripts out of total input transcripts were observed to be annotated with *Arabidopsis* AP2/ERF gene sequences at PLANTTFDB containing important gene definitions (Table 1).

**Table 1:** Prediction and assignments of AP2 GRTs in *W. somnifera* based on *A. thaliana* genome search^29,47^.

NODE_57223_length_1382_cov_9.142547	AP2	AT5G10510.1	1.00E-132	AINTEGUMENTA-like 6
NODE_22774_length_1358_cov_11.654639	AP2	AT2G41710.1	7.00E-93	AP2 family protein
leaf_contig_37257	AP2	AT2G41710.1	1.00E-124	AP2 family protein
leaf_contig_13526	ERF	AT1G64380.1	4.00E-26	ERF family protein
leaf_contig_15277	ERF	AT4G27950.1	2.00E-12	cytokinin response factor 4
leaf_contig_17352	ERF	AT4G39780.1	1.00E-37	ERF family protein
leaf_contig_19337	ERF	AT1G19210.1	4.00E-56	ERF family protein
leaf_contig_20581	ERF	AT1G68550.1	8.00E-16	ERF family protein
leaf_contig_52024	ERF	AT5G13330.1	6.00E-28	related to AP2
leaf_contig_54064	ERF	AT1G19210.1	2.00E-36	ERF family protein
leaf_contig_54131	ERF	AT1G19210.1	1.00E-48	ERF family protein
NODE_17245_length_1038_cov_58.434490	ERF	AT2G47520.1	5.00E-46	ERF family protein
NODE_24041_length_1111_cov_6.091809	ERF	AT1G64380.1	2.00E-72	ERF family protein
NODE_26035_length_1061_cov_6.762488	ERF	AT4G27950.1	2.00E-40	cytokinin response factor 4
NODE_26897_length_1585_cov_132.564667	ERF	AT4G39780.1	7.00E-74	ERF family protein
NODE_46121_length_603_cov_18.933664	ERF	AT2G33710.1	6.00E-38	ERF family protein
NODE_48088_length_163_cov_29.435583	ERF	AT1G72360.2	9.00E-23	ERF family protein
NODE_5893_length_1246_cov_7.693419	ERF	AT1G68550.1	1.00E-23	ERF family protein
NODE_60138_length_225_cov_96.239998	ERF	AT1G19210.1	3.00E-41	ERF family protein
NODE_69997_length_943_cov_7.477201	ERF	AT5G57390.1	1.00E-41	AINTEGUMENTA-like 5
NODE_90464_length_163_cov_87.214722	ERF	AT1G72360.2	4.00E-23	ERF family protein
Root_w_contig_16654	ERF	AT1G22190.1	4.00E-53	ERF family protein
Root_w_contig_2239	ERF	AT2G33710.1	2.00E-29	ERF family protein
Root_w_contig_49312	ERF	AT5G47220.1	3.00E-27	ethylene responsive element binding factor 2
Root_w_contig_57460	ERF	AT5G52020.1	1.00E-43	ERF family protein
Root_w_contig_6893	ERF	AT4G25480.1	1.00E-37	dehydration response element B1A
Root_w_contig_7240	ERF	AT1G19210.1	8.00E-53	ERF family protein

### Gene ontology (GO) assignment

**Figure 3:**
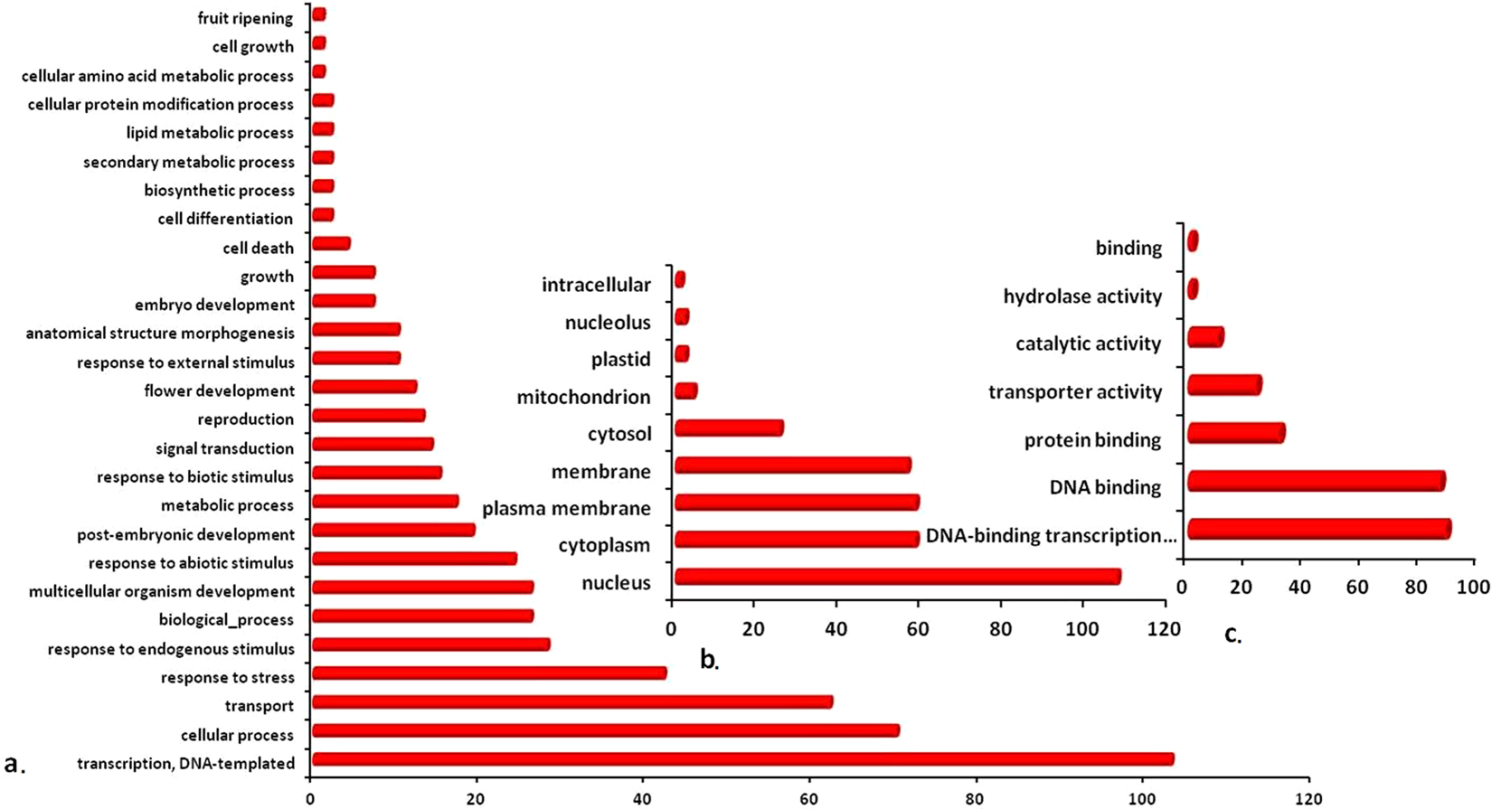
Gene Ontology annotation by assignment of terms for (**a**) Biological process (BP), (**b**) Cellular component (CC) and (**c**) Molecular function (MF) to the whole repertoire (combined transcripts from all the transcriptomes) of AP2 GRTs in *W. somnifera*.

Gene ontology search assigned various terms to the gene transcripts such as cell growth, transport, response to stress, response to stimulus (abiotic, biotic and endogenous), signal transduction, flower and embryo development, secondary metabolic process and so on. Similarly, for metabolic function DNA-binding transcription activity, DNA binding, protein binding and so on were the major terms in addition to the transporter activity. Additionally, for cellular component the major expressed terms were nucleus, cytoplasm, plasma membrane, cytosol and some other terms (Fig. 3).

### Phylogenetic reconstruction of AP2/ERF GRTs in *W. somnifera*

**Figure 4:**
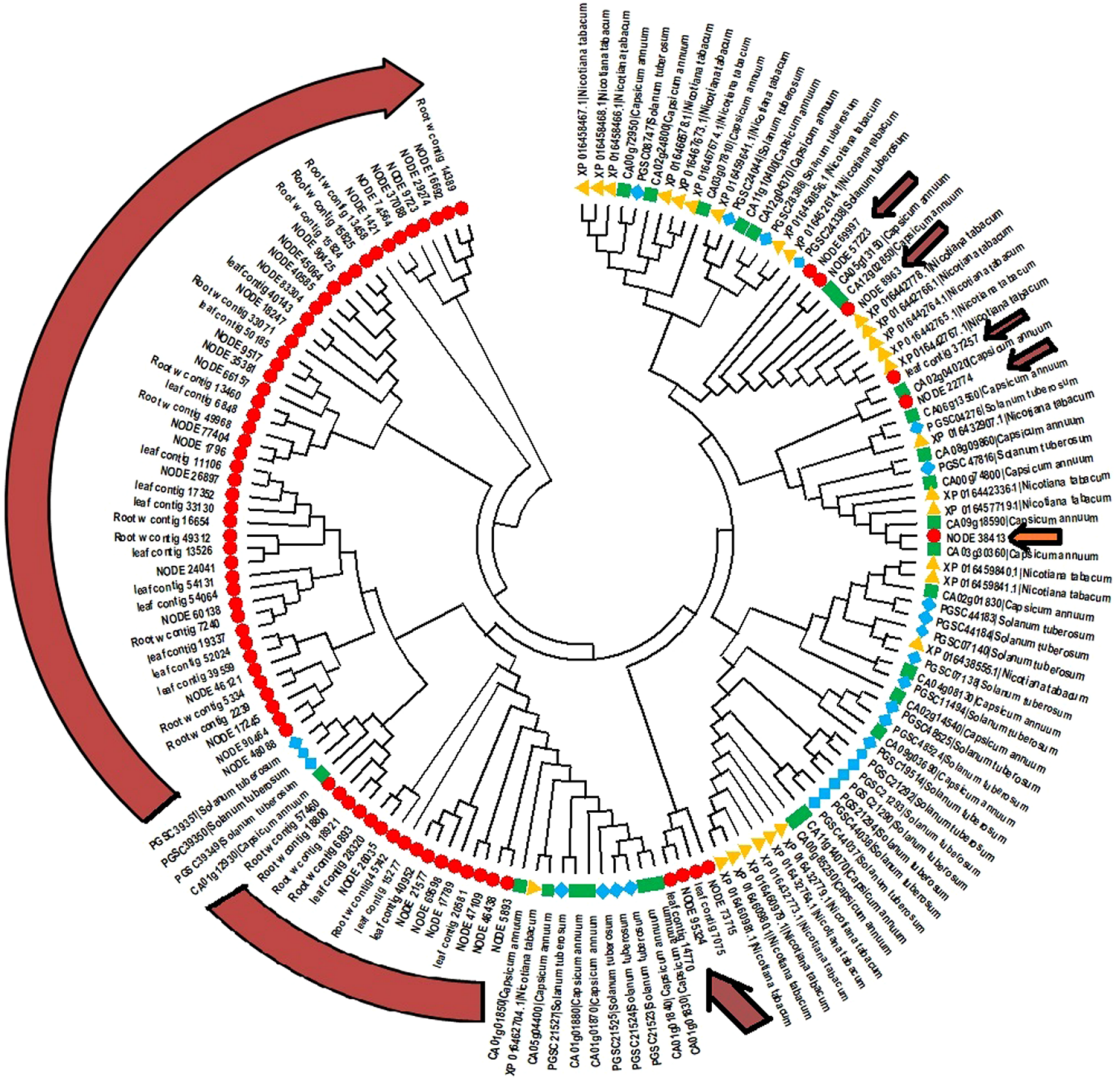
Phylogenetic tree construction for the whole repertoire (combined transcripts from all the transcriptomes) of AP2 GRTs in *W. somnifera* and genes from members of family Solanaceae (*S. tuberosum*, *C. annum* and *N. tabacum*). Node_3814 which was used for further analysis has been separately highlighted by orange color arrow.

Multiple sequence alignment was done using the amino acid sequences of putative AP2/ERF GRTs against proteins. MEGA 6.06 software was used for analysis of the phylogeny and molecular evolutionary pattern (Fig. 4). On analyzing the closeness of AP2/ERF GRTs from *W. somnifera* with *C. annum*, *S. lycopersicum* and *N. tabacum,* 26 transcripts branched as separate groups in which starting from the first group anticlockwise 7 transcripts were classified as separate clades and 18 other transcripts together clubbed in other clade. Root contig 13458 was classified as a separate branch. In another group 21 *W. somnifera* AP2/ERF GRTs were grouped separately but lying in the same clade having 3 AP2/ERF genes from *S. tuberosum*, and 1 related gene from *C. annum* which were closely placed with root contigs 57460, 18800, 18921 and 6893. Leaf contig 28320, Node 26035 and root contig 45742 were branched separately. Similarly, some other transcripts from *W. somnifera* in the same group were observed to form different clades. 12 AP2/ERF GRTs were aligned together with 11 AP2 genes from Solanaceae species containing 6 sequences from *C. annum*, 1 from *N. tabacum* and 4 from *S. tuberosum* (Fig. 4). Node 7315, 95324 and leaf contig 7075, 14770 branched as additional separate groups,. Further, the next two groups mostly were populated with the reference gene sequences from Solanaceae species. Importantly, the AP2/ERF GRTs importantly that aligned with the above mentioned groups were Node 69997, 57223, 8963, 22774, 38413, and leaf contig 37257. Node 38413 was observed to be closely related to *S. tuberosum*, *N. tabacum* and *C. annum* assigned to the same group but branched differently (Fig. 4).

### Conserved motif analysis

**Figure 5:**
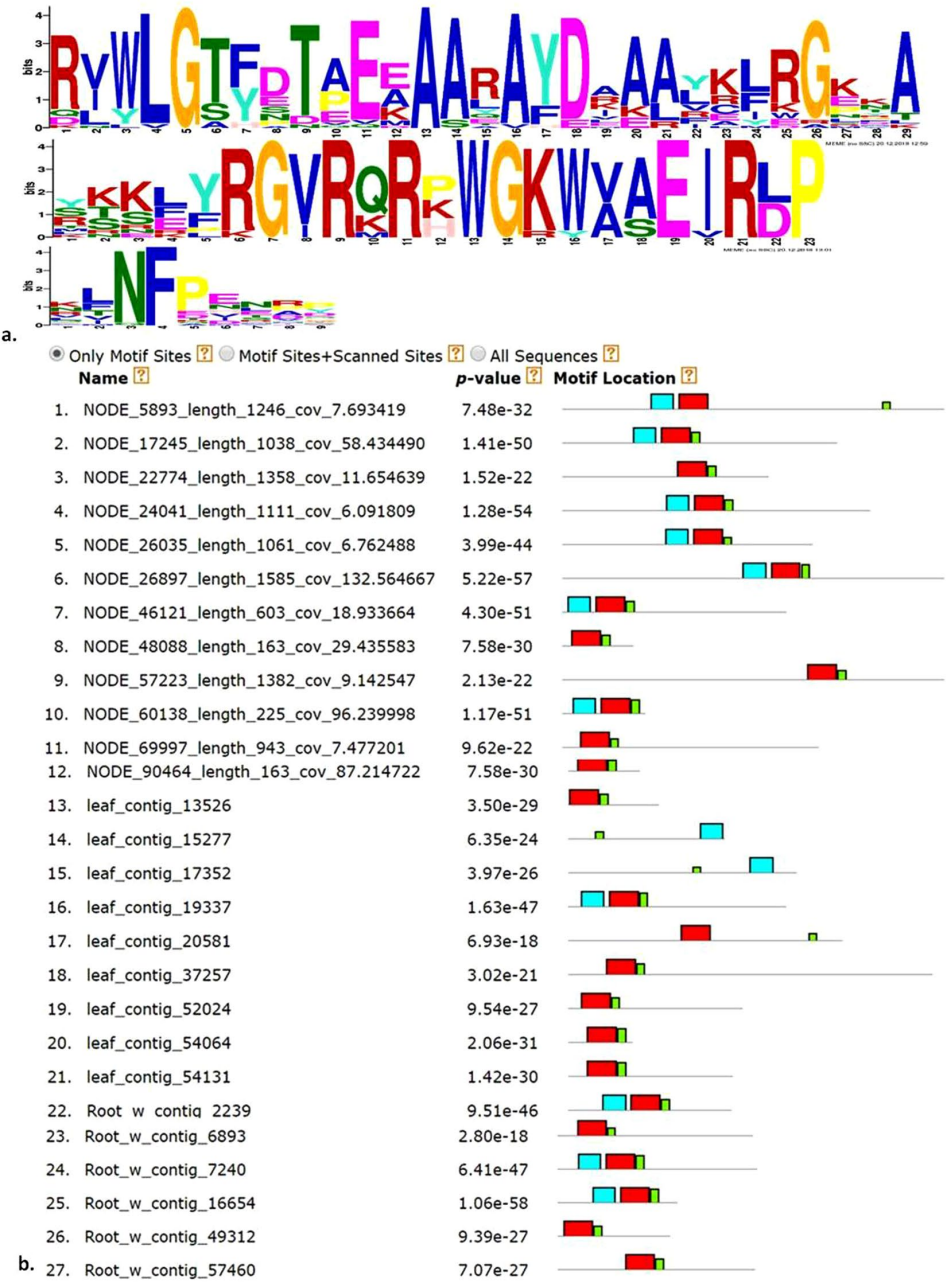
Identification of motifs for the selected AP2 GRTs in *W. somnifera* using MEME suite. (**a**) Sequence logo for three major motifs (**b**) Block diagrams to show the location of the motifs in representative sequences.

The annotated sequences contain enriched motifs for AP2/ERF related transcription factors. The analysis suggested that various AP2/ERF genes contain conserved motifs. Three motifs which were of considerable importance, have been shown for the selected sequences (Fig. 5). Major proportions of *W. somnifera* GRTs showed full AP2/ERF domains while some contained partial domains. The 3 majorly observed motifs were SKKLYRGVRQRPWGKWVAEIRLP as motif 1, AARAYDAAALKLRGKKA as motif 2, and KLNFPENRP as motif 3. The domain importantly present in all the sequences was IPR001471 which has been defined as AP2/ERF domain. The motifs are closely located in some of the sequences while distantly apart in various other sequences (Fig. 5).

### Cloning of *WsAP2*

**Figure 6:**
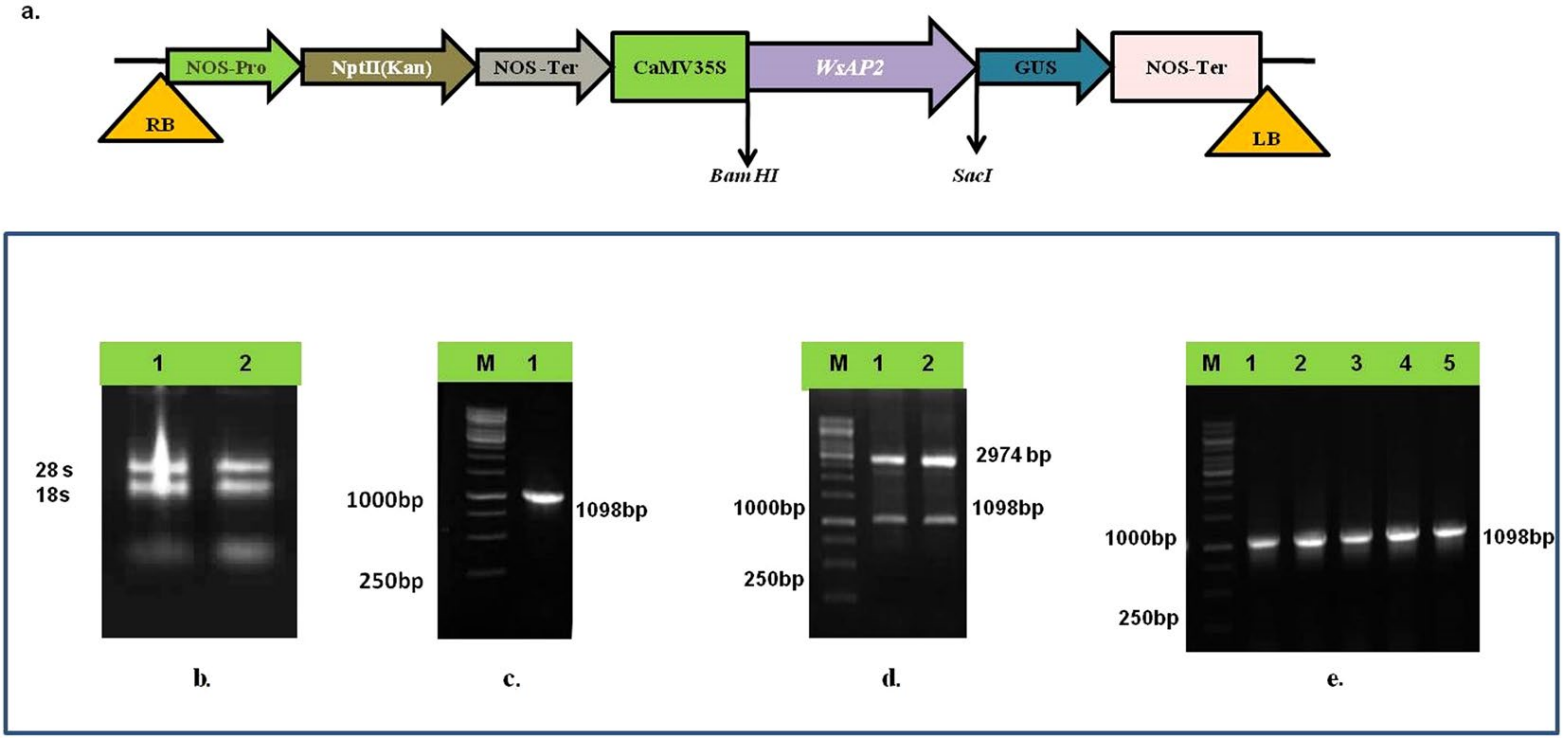
Cloning, restriction digestion, and confirmation of positive clones by colony PCR of full length *WsAP2* (**a**). Recombinant construct map of *pBI121-WsAP2* (**b**).Total RNA isolation from flower of *W. somnifera* (**c**) Amplification of *WsAP2* (1098 bp). (**d**) Restriction digestion of *pJET::WsAP2* clone by respective restriction enzymes for expression in pBI121 binary vector. (**e**) Colony PCR confirmation of cloned *WsAP2*.

**Figure 7:**
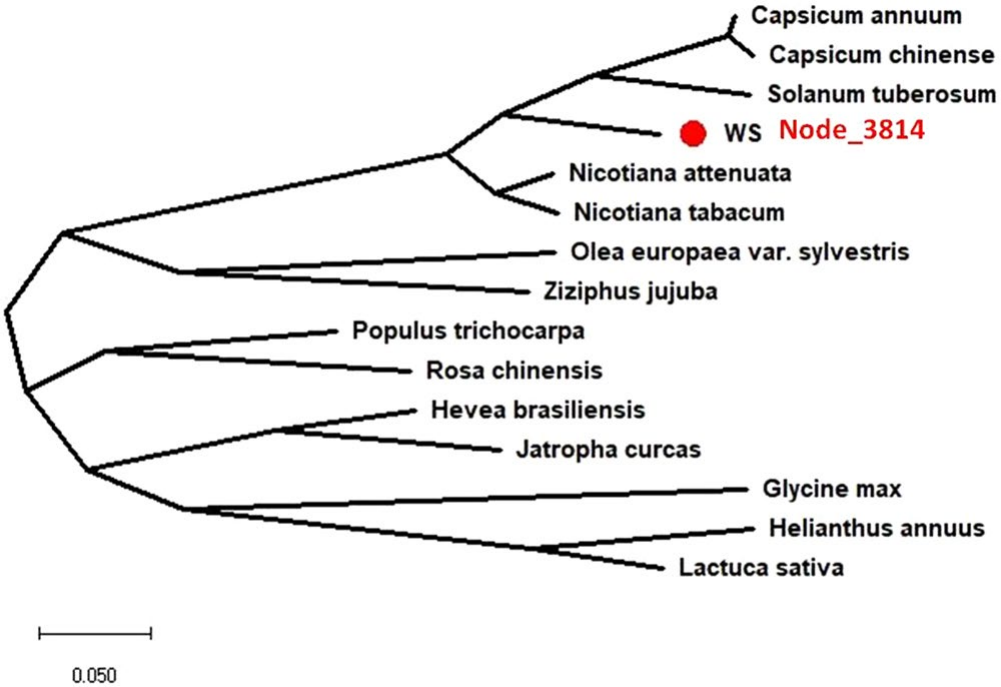
Phylogenetic tree for full length cloned *WsAP2* in *W. somnifera*.

The complete ORF of *WsAP2* was cloned in pJET 1.2 blunt end vector and sequenced (Fig. 6a). The full length (1098 bp) amplicon of *WsAP2* from *W. somnifera* codes for the predicted protein of 366 amino acids (Fig. 6a–e). The calculated mass of WsAP2 protein was 40.2  kDa. Homology matching of *WsAP2* sequence with transcriptomic dataset (Node_3814) confirmed existence of 99 percent sequence identity. The phylogenetic tree constructed for *WsAP2* sequence with the top hit matching sequences during homology search also revealed the closeness of the *WsAP2* gene sequence with other members of family Solanaceae such as *S. tuberosum* (XP_006341423.1), *N. tabacum* (XP_016457719.1), *N. attenuata* (XP_019244135.1), *C. annum* (XP_016562990.1) and *C. chinense* (PHU24180.1) (Fig. 7).

### Tissue specific abundance of *WsAP2* transcripts

**Figure 8:**
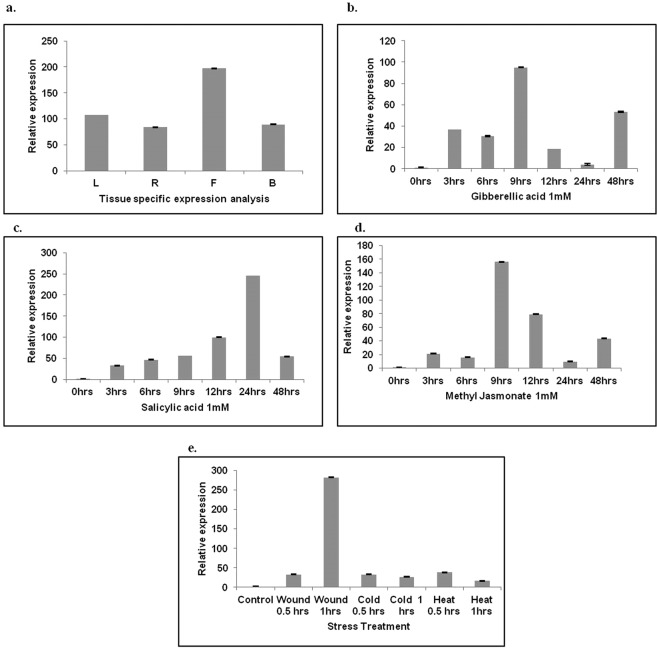
(**a**) Transcript abundance in different tissues of *W. somnifera* (L = Leaf; R = Root; B = Berry and F = Flower). Induced relative expression of WsAP2 gene after elicitor’s treatment (**b**) 1 mM Gibberellic acid (**c**) 1 mM Salicylic acid (**d**) 1 mM Methyl Jasmonate (**e**) Stress (Wounding, Cold and Heat) treatment for 0.5 and 1 hour.

Since expression level of a gene varies across parts of a plant under various biotic and abiotic stresses^37,38^, we analyzed the expression levels of *WsAP2* in different tissues of the *W somnifera* . Our investigations suggested that *WsAP2* was expressed ubiquitously in all parts of *W. somnifera*. Comparative analysis of expression revealed that it was highest in flower followed by leaf. Lowest expression level were recorded in root tissue (Fig. 8a).

### Modulation of *WsAP2* gene expression by signaling molecules and stress treatments

The analysis revealed that the increased expression of the gene was noticed after elicitor (salicylic acid, methyl jasmonate and gibberellic acid), wounding, heat and cold treatments (Fig. 8a–e). In the case of 1 mM gibberellic acid treatment, increment in expression level was recorded after 9 hrs (95 folds) (Fig. 8b).The expression of the gene was upregulated upto 245 folds during the first 24 hrs after treatment with salicylic acid after which down regulation of gene occurred (Fig. 8c). The most profound effect was observed with MeJA treatment with 1 mM concentration where the elevated expression was recorded after 9 hrs of treatment (upto 155 folds) followed by a decreased expression at 12 hrs intervals (Fig. 8d). Wounding increased the transcripts level of *WsAP2* after one hour of treatment (282 folds) while cold treatment reduced the expression of *WsAP2* from 30 min (32 folds) to one hour (26 folds) post treatment. Heat treatment reduced the expression level of transcripts in one hour (16 folds) as compared to 30  min (38 folds) (Fig. 8e).

### Transcript abundance of *WsAP2* and expression level of pathway genes in transiently transformed leaf tissues

**Figure 9:**
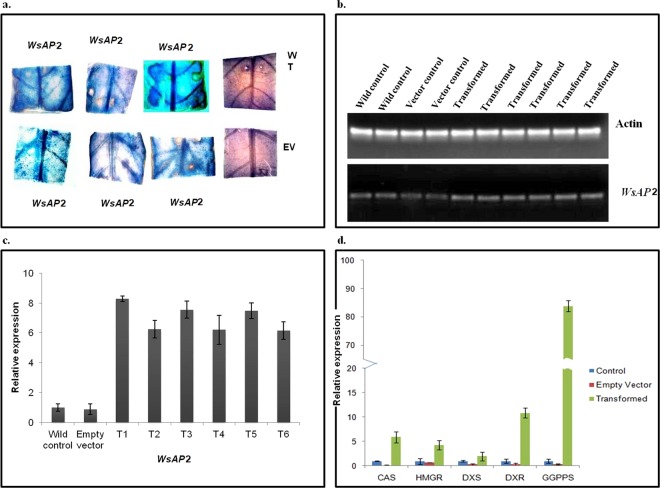
Transient expression of *WsAP2* gene in *W somnifera* leaves with *pBI::WsAP2* construct. (**a**) GUS Histochemical assay in *WsAP2* transformed tissues for confirmation of successful transformation event (**b**) Semi-quantitative gene expression analysis in wild type, vector control and transformed leaves. (**c**) Real time qRT PCR analysis in wild type, vector control and transformed leaves. (**d**) Expression profiling of key genes of MVA and MEP pathway.

The transient transformation assay was chosen for assessment of the functional role of *WsAP2* gene. *WsAP2* was successfully cloned in pBI121 (Fig. 6a,b) and overexpressed in *W. somnifera* leaf explant as indicated by the GUS histochemical analysis (Fig. 9a). Semi-quantitative as well as quantitative real time PCR analysis was done to assess the expression level of *WsAP2* in transformed tissues of *W. somnifera* in comparison with wild type and empty vector control (Fig. 9b,c). Expression levels of transcripts of *WsAP2* in transiently transformed tissues was 6 to 8 times higher in comparison to wild type tissues and empty vector transformed tissues. The data revealed that the elevated expression in transcripts level of *WsAP2* gene in the transgenic tissues, with variation in transcript abundance in different transformed lines.

By using qRT-PCR analysis the expression level of secondary metabolic pathway (terpenoids) related genes like CAS, HMGR, DXS, DXR and GGPPS in transiently transformed tissues of *WsAP2* was also monitored. It was found that all the transcripts showed increased expression in transformed tissues as compared to wild type and empty vector control but the expression of GGPPS was more pronounced (more than 80 folds) than the remaining gene. This result suggested that *WsAP2* may be a master regulator of GGPPS gene and also regulated the MVA and MEP pathway of terpenoid biosynthesis *via* inducing the expression of HMGR, CAS, DXS and DXR gene (Fig. 9d).

## Disscussion

Increasing interest of medicinal plants for therapeutic, neutraceuticals and various other purposes have resulted in the attention of a number of related sectors towards more research on these plants. However, there are many biotic and abiotic+ stresses which these plants have to tolerate posing serious threats to the growth and development of plants. For the exploitation of medicinal plants in different applications and better sustainability, these have to be pest and disease free with improved yield and fewer requirements of fertilizers and water^39,40^. Environmental factors and pathogens have strong impact on plants during its development. Since AP2/ERF genes play significant role in plant development and various stress responses, whether biotic or abiotic, these present ideal candidature for investigating the regulatory mechanism of related process. The improved tolerance for biotic and abiotic stress requires special focus on plants under the physiological and molecular mechanism towards these stresses as for example in *W. somnifera*. The significance of the objective to identify and isolate AP2/ERF GRTs is towards understanding the molecular genetic basis which would facilitate the improvement of *W. somnifera* and provide the functional genetic resource meant for transgenic research. Transcription factors are very important in modulation of acclimatization of plant responses towards different external or internal cues. These significantly govern downstream gene expression in response to stress exposure *via* gene activation/repression in case of stress and signal transduction pathways. These are present in the plant genome in large numbers. In our previous study, we have demonstrated the presence of significant proportion of transcription factors in *W. somnifera*^29^. On the basis of conserved AP2-related domains AP2/GRTs were identified to be grouped as AP2/ERF superfamily members in *W. somnifera* transcriptome in this analysis. The availability of datasets for various plants at Plant (TFDB) facilitated the identification and comparison of families and groups at default parameters. The annotation as predicted, based on comparison with various databases (db) such as Arabidopsis genome db, Interpro db and TFCAT db confirmed the presence AP2/ERF domains in the sequences and thus suggests the correctness of annotation *via* these databases. The results of selected AP2/ERF genes through phylogenetic analysis were observed to be in confirmation with the results of whole transcripts phylogenetic comparison with other Solanaceae species members, as the selected Node_3814 was aligned close to the *Nicotiana sp., S. tuberosum, Capsicum sp*. Not every *W. somnifera* AP2/ERF gene has counterpart in *N. tabacum*, *S. tuberosum*, *C. annnum* depicting the probability that *W. somnifera* had undergone differential expansion separately. The candidate transcripts for AP2/ERF proteins were used in MEME motif analysis for identification of conserved motifs in families and subfamilies. The results from conserved motif analysis may provide leads to further classify the putative candidates, as identical motifs are likely to have similar function^41^.

Various transcription factors were cloned and characterized from *W. somnifera* but the role of *AP2-ERF* TF was still not understood. For the assesment of the role of AP2-ERF transcription factor in *W. somnifera via in-silico* methods followed by *in-planta* validation, this study was carried out.The transient overexpression study of *WsAP2* showed that this gene was successfully transformed in *W. somnifera*. It was already known that AP2 transcription factor was involved in growth and development of plant as well as in the regulation of biotic and abiotic stress response^3,42^. It was reported that salicylic acid and jasmonic acid are important activators of defence related genes in plant system. In addition to this plant hormone gibberellic acid regulates the crosstalk of different signaling cascades occurring during different abiotic stress responses^43^, such as drought, salt, and cold. In our current study, the transient overexpression of *WsAP2* was found to be induced not only by salicylic acid, methyl jasmonate and gibberellic acid but also by abiotic stresses like wound, heat and cold treatments. This has led to the conclusion that *WsAP2* might act as a connecting link between different signalling pathways abiotic stress responses in plant. Jasmonic acid is involved in the rearrangement of gene expression of secondary metabolism in response to various types of environmental and developmental stimuli. Thus, jasmonic acid is a strong inducer of secondary metabolism^44^. Several jasmonate (methyl jasmonate) inducible AP2 transcription factors were studied to be involved in the regulation of secondary metabolic pathway related enzymes. For example overexpression of *AaERF1* or *AaERF2* were associated with increased accumulation of artemisinin and artemisinic acids^45,46^. In our study, it was found that after transient overexpression of *WsAP2* in plant, the expression levels of secondary metabolism related genes like CAS, HMGR, DXS and DXR was increased and GGPPS were maximally induced after *WsAP2* overexpression confirming the involvement of *WsAP2* in terpenoid metabolism.

## Supplementary information

Table S1.
