# Supplementary material for: In silico mining and functional analysis of AP2/ERF gene in Withania somnifera
Source: Sci Rep. 2020 Mar 17;10:4877. doi: 10.1038/s41598-020-60090-7 (PMC7078187; doi:10.1038/s41598-020-60090-7)
Supplement: Supplementary file 1 — Table S1. [file 41598_2020_60090_MOESM1_ESM.doc]

***In silico* mining and functional analysis of AP2/ERF gene in *W. somnifera***

**Sandhya Tripathi^ab#^, Yashdeep Srivastava^a#^, Rajender Singh Sangwan^abc^, Neelam Singh Sangwan^abc*^**

^a^Department of Metabolic and Structural Biology, CSIR-Central Institute of Medicinal and Aromatic Plants (CSIR-CIMAP), Lucknow 226015, India

^b^Academy of Scientific and Innovative Research (AcSIR) (*An Institution of National Importance by Act of Parliament*), CSIR-HRDC Campus, Kamla Nehru Nagar, Sector-19, Ghaziabad-201002, UP, India

^c^Department of Biochemistry, School of Interdisciplinary and Applied Life Sciences,Central University of Haryana, Jant-Pali, Mahendergarh Haryana 123031, India

^#^ Both the authors contributed equally in the manuscript

**Correspondence**

*Corresponding author,

e-mail: [nsangwan5@gmail.com](mailto:nsangwan5@gmail.com)

**Table S1. Primers used in the study**

| **Primer Name** | **Sequence** |
| --- | --- |
| **Act FP** | 5’- CTTTCTACAATGAGCTTCGTG-3’ |
| **Act RP** | 5’- ATACAGTGAGAGAGGACAGCCTG-3’ |
| **nptII F** | 5’-CTGAATGAACTGCAGGACGAGG-3’ |
| **nptII R** | 5’-GCCAACGCTATGTCCTGATAGC-3’ |
| **GUS F** | 5’-CTGTGGAATTGATCAGCGTTGGTGG-3 |
| **GUS R** | 5’-CCTGATGCTCCATCACTTCCTGAT-3 |
| **WST11FLR** | 5’-GAATTCCTAGCAATCAAGCTCACACTG-3’ |
| **WST11FLF** | 5’-GGATCCATGGCTAAAACATCAAAGTCG-3’ |
| **WSTF11FLpBIF** | 5’-TCTAGAATGGCTAAAACATCAAAGTCG-3’ |
| **WSTF11FLpBIR** | 5’-GGATCCCTAGCAATCAAGCTCACACTG-3’ |
| **WST11RTF** | 5’-GGAGCTAAAGGAAATGGAAG-3’ |
| **WST11RTR** | 5’-CCTCTTCAGTAGCATATGTTC-3’ |
